# Flood resilience in paired US–Mexico border cities: a study of binational risk perceptions

**DOI:** 10.1007/s11069-022-05225-x

**Published:** 2022-02-09

**Authors:** Christopher A. Freimund, Gregg M. Garfin, Laura M. Norman, Larry A. Fisher, James L. Buizer

**Affiliations:** 1 School of Natural Resources and the Environment, University of Arizona, Tucson, AZ USA; 2 U.S. Geological Survey, Western Geographic Science Center, Tucson, AZ USA

**Keywords:** Flooding, Risk perception, Transboundary, US–Mexico border, Resilience, Green infrastructure

## Abstract

Disastrous floods in the twin cities of Nogales, Arizona, USA, and Nogales, Sonora, Mexico (collectively referred to as Ambos Nogales) occur annually in response to monsoonal summer rains. Flood-related hazards include property damage, impairment to sewage systems, sewage discharge, water contamination, erosion, and loss of life. Flood risk, particularly in Nogales, Sonora, is amplified by informal, “squatter” settlements in the watershed floodplain and associated development and infrastructure. The expected increase in precipitation intensity, resulting from climate change, poses further risk to flooding therein. We explore binational community perceptions of flooding, preferences for watershed management, and potential actions to address flooding and increase socio-ecological resilience in Ambos Nogales using standardized questionnaires and interviews to collect data about people and their preferences. We conducted 25 semi-structured interviews with local subject matter experts and gathered survey responses from community members in Ambos Nogales. Though survey response was limited, expected frequencies were high enough to conduct Chi-squared tests of independence to test for statistically significant relationships between survey variables. Results showed that respondents with previous experience with flooding corresponded with their level of concern about future floods. Additionally, respondents perceived greater flood-related risks from traveling across town and damage to vehicles than from inundation or damages to their homes or neighborhoods. Binationally, women respondents felt less prepared for future floods than men. On both sides of the border, community members and local experts agreed that Ambos Nogales lacks adequate preparation for future floods. To increase preparedness, they recommended flood risk education and awareness campaigns, implementation of green infrastructure, additional stormwater infrastructure (such as drainage systems), enhanced flood early warning systems, and reduction of flood flows through regulations to reduce the expansion of hard surfaces. This study contributes systematic collection of information about flood risk perceptions across an international border, including novel data regarding risks related to climate change and gender-based assessments of flood risk. Our finding of commonalities across both border communities, in perceptions of flood risk and in the types of risk reduction solutions recommended by community members, provides clear directions for flood risk education, outreach, and preparedness, as well as measures to enhance cross-border cooperation.

## Introduction

Global climate models project profound changes in the climate of the US–Mexico border. Compared with the 1971–2000 historical record, the projections include as much as a 20% decrease in annual precipitation, 4–5 °C increases in annual average temperatures, by 2041–2070, and increases of a month or more of days with maximum daily temperatures greater than 38 °C (100°F) (Wilder et al. 2013); these changes will lead impacts of varying severity, depending on differences in the on socio-economic status of border communities. This borderland region reflects distinctive social, political, and environmental issues, including climatic extremes of heat and drought, water quantity and quality issues, land-use change, rapid population growth, human migration, drug trafficking and militarization of the border, and social and environmental impacts of the newly constructed/reinforced border wall (Freimund 2020; Lara-Valencia and Diaz-Montemayor 2010; Norman et al. 2012a; Wilder et al. 2020; WRI 2020).

**Fig. 1:**
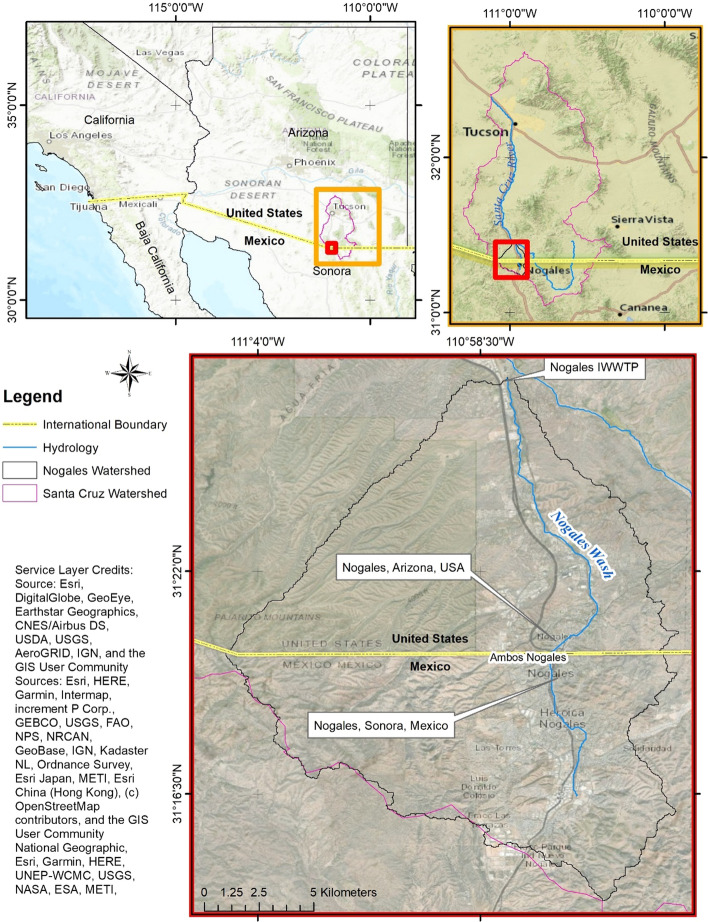
Maps portraying the location of the Ambos Nogales watershed, the Nogales Wash, the twin cities of Nogales, Arizona, and Nogales, Sonora, Ambos Nogales, in relationship to the Santa Cruz River and watershed along the US–Mexico Border and proximity to Tijuana, Baja California, Mexico

Along the Arizona-Sonora border lie the sister cities of Nogales, Arizona, and Nogales, Sonora (Ambos Nogales; Fig. 1). Water flows from the southern tip of the Nogales watershed, across the US–Mexico border and through the twin cities. These binational communities face an array of border-related issues as well as frequent powerful and lethal floods (Huth and Tinney 2008; Kelly-Richards and Banister 2017; Lara-Valencia and Diaz-Montemayor 2010; Magaña et al. 2012; Márquez Reyes 2010; Norman et al. 2010a, b; U.S. Army Corps of Engineers 2004; Wilder et al. 2012). Nogales, Sonora, faces rapid population growth and urbanization (Norman et al. 2012b; Wilder et al. 2012), growing development in the floodplain (Norman et al. 2009, 2010a), and replacement of the natural environment with built infrastructure (Norman et al. 2010b, 2012b). The combination of population growth, urbanization, variation in precipitation, and climate change may increase flood intensity and heighten flood risk to those living on both sides of the watershed (Wilder et al. 2012).

Our goal was to understand perceptions of risk and to identify strategies to increase resilience to flooding throughout the Nogales watershed. Key informants from Ambos Nogales provided feedback and suggestions about the research questions and associated methodologies to make them as relevant and as accessible as possible to the communities. We aimed to answer the following three research questions through a series of interview and survey questions:

1. What are the perceptions of flood risk throughout the binational communities of Ambos Nogales?
2. How do these perceptions vary throughout the binational watershed?
3. What are the preferences for flood management in the binational Nogales watershed and what strategies or actions are being developed to increase resilience to flooding or to adapt to future flood risks at the municipal and binational watershed levels?

### Flood risk perception

Flood risk can be defined as a combination of three components: the flood itself (intensity and likelihood), exposure (potential damages to people or property), and vulnerability (community or regional susceptibility) (Jongman et al. 2015; Kron 2005; Tanoue et al. 2016). A socio-environmentally vulnerable population, by definition, has lower education or less access to resources, which poses disparities in environmental risks and burdens and limits their adaptive capacity (Norman et al. 2012c). Flood risk increases with more intense or numerous floods, with increased urbanization in the floodplain, or increases in socio-environmental vulnerability (Kron 2005; Norman et al. 2012b).

Flood risk perception depends on an individual’s understanding of potential flood impacts and the probability of their occurrence (Bradford et al. 2012; Lechowska 2018; Raaijmakers et al. 2008). Flood risk perception is often categorized as a combination of awareness of the hazard and its impacts, preparedness for future floods at the city or individual level, as well as concern about future floods (Bradford et al. 2012; Lechowska 2018; Raaijmakers et al. 2008; Scolobig et al. 2012). Awareness refers to knowledge about floods and their impacts, which often increases with previous flood experience (Bradford et al. 2012; Brilly and Polic 2005; Franklin et al. 2014; Scolobig et al. 2012; Terpstra 2011). Flood preparedness includes actions or strategies taken through official authorities or social networks at the household or city level to reduce potential future impacts (Bodoque et al. 2016; Burningham et al. 2008; Lutoff et al. 2016; Scolobig et al. 2012; Terpstra 2011). As discussed by Bradford et al. (2012), worry or concern is a crucial connection between awareness and preparedness. Someone may be aware of the floods, but not implement preparedness measures if they are unconcerned (Bradford et al. 2012). Flood risk perception is also connected to local value systems and the level of trust in governmental or structural mitigation actions such as levees (Morris-Oswald and Sinclaire 2005; Scolobig et al. 2012; Lechowska 2018). Increased levels of trust in flood protection strategies or institutions may decrease concern, signifying a decrease in risk perception. (Bradford et al. 2012; Scolobig et al. 2012; Terpstra 2011). Lack of knowledge about the hazard may decrease concern, limiting risk perception (Scolobig et al. 2012; Lechowska 2018). Lower levels of concern, due to increased levels of trust in local institutions, flood protection infrastructure, or the belief that one will be unaffected, may decrease individual preparatory actions (Burningham et al. 2008; Bradford et al. 2012; Scolobig et al. 2012; Terpstra 2011).

### Transboundary flood management

The transboundary context increases the challenges associated with managing the impacts of floods. The USA and Mexico have very different governing structures; data quality and quantity differ, jurisdictional entities vary substantially, and the two nations’ financial capacities also differ (Bakker 2009; Norman et al. 2006; 2012c; Varady and Mack 1995; Varady et al. 2020; Wilder et al. 2012). In Mexico, water is controlled and managed by a federal entity, CONAGUA (Norman et al. 2013; Tapia‐Villaseñor et al. 2020; Varady et al. 2020; Wilder et al. 2013; Wilder et al. 2020). In contrast, in the USA, water is managed in a decentralized system (Tapia‐Villaseñor et al. 2020; Varady et al. 2020; Wilder et al. 2020) in which each state, water district, and management area have their own rules and regulations. The differences in the US and Mexico systems complicate binational collaboration on shared plans for cross-border water, wastewater, or hazard management (Scott et al. 2012; Wilder et al. 2013). Thus, binational actions to increase resilience must navigate asymmetries in governance or make the most of informal governance through non-governmental collaborations (Scott et al. 2012; Tapia‐Villaseñor et al. 2020; Wilder et al. 2013). We hypothesized that the variance in country of origin and different socio-economic classes would impact, if not dictate, local perceptions. Understanding this transboundary system, and the flood risk perceptions in communities throughout this binational watershed, is key to creating impactful resilience strategies (Raaijmakers et al. 2008).

### Institutional context

Bakker (2009) showed that globally, there are very few transboundary institutions established to solely address flooding issues; none of these are in the Americas. Although not primarily created to address flooding, in the US–Mexico border region, the International Boundary and Water Commission (IBWC) and La Comisión Internacional de Límites y Aguas (CILA) form the binational institutional structure that manages water agreements (GNEB 2016; IBWC 2012; Tapia‐Villaseñor et al. 2020; Varady et al. 2020; Wilder et al. 2020). These agreements include managing water allotments, sanitation and water quality, wastewater treatment, and flood control (GNEB 2016; IBWC 2012; Tapia‐Villaseñor et al. 2020). In 2010, the IBWC/CILA funded a US Geological Survey study to document the impacts of gabions (wire caged dams, filled with rocks to allow water to slowly flow through) installed in the headwater tributaries of Ambos Nogales to reduce flooding (Norman et al. 2010b).

IBWC/CILA is a joint institution, with federal US and Mexican representatives, that facilitates agreements, treaties, and Minutes. Minutes are official technical amendments to the 1944 Treaty regarding border and water issues, signed by the IBWC commissioners from both the USA and Mexico (IBWC 2012). Wilder et al. (2020) claimed that the Minutes offer flexibility to enact new solutions within the IBWC formal treaty structure. Flood control is an original function of the 1944 Water Treaty and there is a precedent of passing Minutes relating to binational flooding and stormwater issues. For example, in the Tijuana Basin, this includes Minutes: 225, 236, 258, and 320 (IBWC 1944, 2012; Mumme 2019; Pineda Pablos et al. 2020). Minute 320 created a binational framework and a binational core working group, comprised of governmental and non-governmental organizations, to jointly create plans and implement projects throughout the watershed; the plans and projects include flood management, stormwater management, and sediment and trash management (IBWC 2015; Mumme 2020; Pineda Pablos et al. 2020). Since 1958, the IBWC has passed multiple Minutes focused on Ambos Nogales (Tapia‐Villaseñor et al. 2020) related to sewage wastewater management. The Nogales International Wastewater Treatment Plant (NIWTP) is managed by IBWC/CILA in Arizona and treats a large portion of the sewage from Nogales, Sonora, Mexico, which retains ownership of the treated effluent (Norman et al. 2013; Tapia‐Villaseñor et al. 2020; Varady et al. 2020). In 2017, stakeholders from both nations met with the IBWC commissioners to discuss the long history of binational flooding in Ambos Nogales and proposed the idea of creating a Minute focused on stormwater management in the Nogales watershed (IBWC 2017). The Commissioners agreed to develop the Minute, after reviewing stormwater data and hydraulic modeling and monitoring assessment evidence presented by the USGS, US Department of Agriculture (USDA), and the National Weather Service; since then, the presiding Commissioners have retired, and the Minute has not yet been created (IBWC 2017). Although there is not an official IBWC working group focused on flooding, there is a history of cross-border collaborative networks that include governmental agencies such as the Arizona Department of Environmental Quality (ADEQ) and non-governmental organizations focused on shared environmental and water issues, including the Sonoran Institute, Watershed Management Group, and Friends of the Santa Cruz River (Scott et al. 2012; Wilder et al. 2013).

### Ambos Nogales

The border communities of Ambos Nogales are located approximately 105 km south of Tucson, Arizona, USA, in the Sonoran Desert (Fig. 1). They are the largest border communities on the Arizona-Sonora border (Tapia‐Villaseñor et al. 2020). In the 2010 census, around 21,000 people lived in Nogales, Arizona. Population estimates for Nogales, Sonora, at that time ranged from 200,000 to over 350,000 people (Norman et al. 2012a; Tapia‐Villaseñor et al. 2020). The Nogales Wash is a tributary to the binational Santa Cruz River, which begins in Arizona, USA, flows south into Sonora, Mexico, and returns to Arizona, eight kilometers east of Ambos Nogales (Huth and Tinney 2008; Norman et al. 2006, 2010a; Wilder et al. 2012). The water in the Nogales watershed generally flows from south to north based on topography (Huth and Tinney 2008; Norman et al. 2010a). In the southern portion of the watershed, the topography is steep and hilly, and construction of homes and neighborhoods has largely taken place on these hills, replacing the natural environment with built infrastructure (Norman et al. 2010a; Wilder et al. 2012). The binational urban center is in a narrow valley, within the floodplain (Huth and Tinney 2008; Norman et al. 2010a; Wilder et al. 2013). The border wall cuts across the center of the Ambos Nogales watershed, separating the two downtown areas and creating a physical barrier where people and water must cross from one country to the other (Norman et al. 2010a). These twin cities are located in a semi-arid desert environment, characterized by highly variable precipitation (Wilder et al. 2012). Average annual precipitation in Ambos Nogales is 18 inches (460 mm), more than half of which falls during the summer monsoon season, the rest falling during a winter rainy season (Kelley-Richards and Banister 2017; Wilder et al. 2013).

Flooding events in Ambos Nogales have caused damage to infrastructure, destruction of property, and loss of life (Lara-Valencia and Diaz-Montemayor 2010; Marquez-Reyes 2010; Norman et al. 2010a; Wilder et al. 2012). Floods have affected Ambos Nogales for decades, including notable floods in 1957, 1983, and 2008 (Márquez Reyes 2010; Norman et al. 2010a). The geographic setting of Ambos Nogales, combined with the summer monsoon season, can cause dangerous conditions for residents living in downtown floodplains, downstream from precipitation (Kelley-Richards and Banister 2017). Flood danger has been exacerbated by the development of urban and border infrastructure, such as the border wall, which has impeded flows, causing disproportionate damage in Nogales, Sonora (Lara-Valencia and Diaz-Montemayor 2010; Marquez-Reyes 2010). Flooding can also lead to public health issues caused by sewage discharge and leaky pipes associated with the NIWTP conveyance within the Nogales Wash (Norman et al. 2012a, c). Many families living within Nogales, Sonora, lack access to water and sewage infrastructure; thus, contamination from private sewage systems occurs during floods (Norman et al. 2006, 2010a, 2012a, c; Kelley and Bannister 2017; Varady and Mack 1995; Wilder et al. 2012).

Various interconnected factors, including rapid population growth and socio-economic inequities lead to Ambos Nogales’ vulnerability to flooding and other impacts of climate change (Scott et al. 2012; Norman 2005; Norman et al. 2006, 2008a, b; Norman and Wallace 2008; Norman et al. 2012c; Wilder et al. 2013, 2020). The population of Ambos Nogales has grown rapidly, particularly Nogales, Sonora. (Norman 2005; Norman et al. 2008a, 2009). This is primarily a result of increased employment opportunities through large-scale industrial production facilities known as *maquiladoras* (Granados‐Olivas et al. 2016; Ingram and Varady 1994; Norman et al. 2006; Scott et al. 2012). The Immigration Reform and Control Act and the North American Free Trade Agreement (NAFTA) stimulated growth and industrialization on the border, causing Nogales, Sonora, to become a hub of the *maquila* industry (Granados‐Olivas et al. 2016; Norman et al. 2006; Scott et al. 2012; Wilder et al. 2020). Much of this growth has taken place in informal and unincorporated communities known as *colonias*; many *colonias* lack access to adequate housing and services, including sewage systems and potable water (Norman et al. 2006, 2012a; Wilder et al. 2012).

## Methods

To investigate our three research questions, Freimund (2020) conducted an initial round of semi-structured interviews with local experts, a binational electronic survey with community members, and a second round of follow-up interviews with subject matter experts. The first round of interviews aimed to better understand the causes and impacts of floods, the institutional context, and suggestions for reducing flood risk throughout the watershed. Next, a binational survey was developed, using the Social Values for Ecosystem Services (SolVES) instrument, developed by the US Geological Survey (Sherrouse et al. 2011, 2014; Petrakis et al. 2020). This survey was designed to investigate community flood risk perception and preferences for flood management. Finally, to corroborate the survey findings and gain a deeper understanding of risk perception in Ambos Nogales, a second round of interviews with local water resource professionals was conducted. This iterative approach allowed for the interviewees to reflect on the barriers, opportunities, and perceptions of survey participants to make the most of public sentiment and reinforced their involvement in the process. These methods are described in depth below.

### Initial interviews

**Fig. 2:**
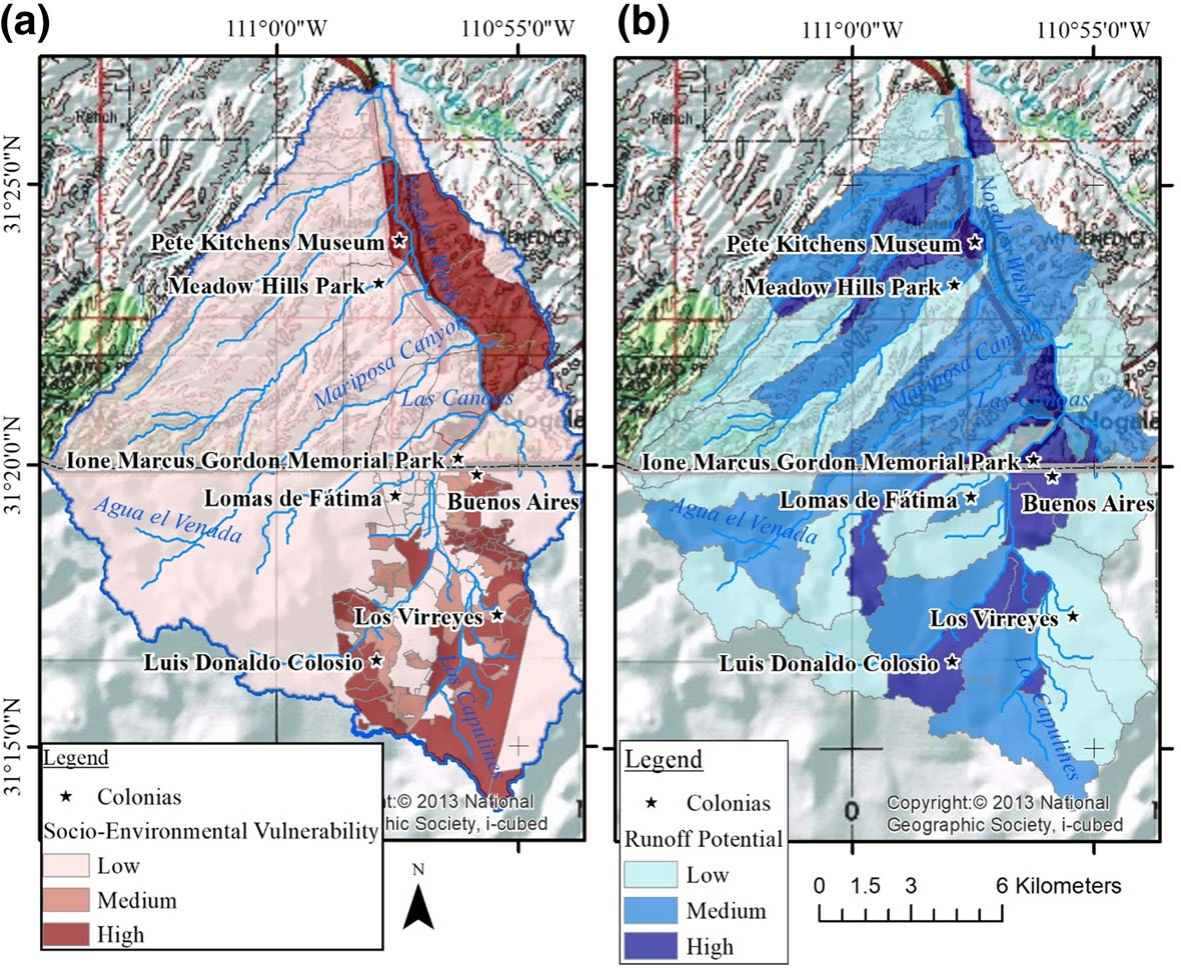
Choropleth maps of in the Ambos Nogales watershed displaying **a** the geometrical interval distribution of the M-SEVI to identify areas of high, medium, and low socio-environmental vulnerability (modified from Norman et al. 2012a) and **b** watershed runoff (mm) classified into three categories of runoff to identify areas of high, medium, and low risk to potential flooding (modified from Norman et al. 2010a)

Freimund (2020) conducted in person and remote semi-structured qualitative interviews with local water resource professionals and subject matter experts. A snowball sampling methodology was employed, where each interviewee identified other potential participants, until no new names were identified (Given 2008). These interviews provided a deeper understanding of the causes of flooding, identified priority actions or strategies to increase resilience, discussed collaboration among key actors in Ambos Nogales, and analyzed barriers to implementing these actions. Thirty-six experts were invited to participate, of whom 14 accepted interviews; nine interviews were conducted in Arizona and five in Sonora. Interviewees included federal, state, and municipal personnel in Arizona and Sonora, as well as county personnel in Arizona, and representatives from a non-governmental environmental organization. Responses from the initial interviews were categorized by key question and gathered into themes, based on repeated responses (Freimund 2020). Interview themes informed the flood-related survey questions, which examined flood concern, perceptions of personal preparedness, city preparedness, and willingness to pay or volunteer for improving flood management (Fig. 2).

### Social values for ecosystem services (SolVES)

The SolVES was designed to use participatory mapping and questions about stakeholder preferences to understand and map the connection between social values and ecosystem services (Sherrouse et al. 2011, 2014; Petrakis et al. 2020). Freimund (2020) is the first to use this tool in a binational setting and developed a bilingual digital version of the survey for this research. In addition to the mapping of social values, the survey instrument included questions about community members’ experiences with flooding and preferences for future flood management strategies. Our initial sampling plan was to implement the survey using a stratified sample of potential vulnerabilities of the neighborhoods within the watershed boundary. We based this on the Modified Socio-Environmental Vulnerability Index (M-SEVI) that used determinants from binational census and neighborhood data describing levels of education, access to resources, migratory status, housing, and number of dependents, to portray the region’s populace (Norman et al. 2012a; Fig. 2a). We also selected areas prone to different levels of risk related to stormwater runoff (Norman et al. 2010a; Fig. 2b). Utilizing these vulnerability indexes in combination, we compared the colonias’ (denoted by stars) level of socio-environmental vulnerability with their risk to runoff potential (Fig. 2a, b); drawing on suggestions from local subject matter experts, we selected seven neighborhoods/colonias throughout the binational watershed, corresponding to low, medium, and high vulnerability levels in both Nogales, Arizona, and Nogales, Sonora (Fig. 2).

We started by conducting the survey with groups of parents whose children were students in school in our selected neighborhoods. This method was suggested to us by Cooperative Extension personnel and other local contacts to reach similar groups of participants on both sides of the border. We conducted the survey from January–March 2020 in Nogales, Arizona. We began the process of selecting institutions to survey in Nogales, Sonora, the COVID-19 pandemic began. With social distancing restrictions in place, we altered our survey sampling methodology to be completely remote. From March–May 2020, we used email and text messaging to send links to Spanish and English language versions of our survey. We conveyed the messages to colleagues in the area, local environmental list-servs, and to local businesses and churches; we selected the businesses and churches, based on the public availability of their contact information in both Arizona and Sonora. It is therefore important to underscore that the respondents did not constitute a random sample, as there was no control or stratification of survey participants once it was distributed electronically. Despite this limitation, the survey and subsequent analysis demonstrate the potential for utilizing this instrument in a binational setting during a global pandemic. With these limitations in mind, we conducted follow-up interviews with experts to corroborate the survey results and gain further insights into the response data (Tables 1 and 2).

**Table 1:** Survey responses within the Nogales watershed, separated by self-identified gender. Adapted from Freimund (2020)

	Country			Total
	USA	Mexico	Did not specify	
*Gender*				
Men	15	25	3	43
Women	35	12	10	57
Did not specify	6	11	3	20
Total	56	48	16	120

**Table 2:** Survey responses within the Nogales watershed, separated by vulnerability level Adapted from Freimund (2020)

Country	Low (n)	Medium (*n*)	High (*n)*	Did not specify neighborhood	Total
USA	32	9	10	5	56
Mexico	28	11	9	0	48
Did not specify	0	0	0	16	16
Total	60	20	19	21	120

The survey included questions within the following thematic groupings suggested by interviewees and drawn from flood risk perception literature (Bradford et al. 2012; Lechowska 2018; Raaijmakers et al. 2008; Scolobig et al. 2012): Flood Concern, Previously Experiencing Flood Impacts, Perception of Future Impacts, Perceptions of Personal Preparedness, Individual Actions, Perceptions of City Preparedness, and Willingness to Pay or Volunteer for improved flood management. Relationships between survey variables were explored and tested for significance using the Chi-squared test with the statistical software STATA. For example, relationships between personal preparedness (theme) and the survey variables home ownership, gender, and perceptions about future impacts could then be examined. Although a higher number of responses would give more reliable statistical assurance for hypothesis testing, the Chi-squared statistic tests for a relationship between categorical variables do not require a large number of respondents to be conducted. The key findings were then presented to local experts during the follow-up interviews.

#### Survey sample bias

Eight surveys were sent to every personal email in the watershed, in addition to some emails that were harvested from Internet searches for community organizations and participants were asked to forward the questionnaire to all of their contacts. We received 185 survey responses, 120 of which stated that they lived within the Nogales watershed. We relied on local contacts throughout the watershed to distribute the link within their networks. We sent out two reminder emails to the same list of contacts who then re-sent the URL. Due to the anonymity of the survey, we were unable to track response rate once emailed out to colleagues. Because we were unable to pursue our original sampling plan to stratify questionnaires to varying vulnerability sectors, we received more responses from communities with lower M-SEVI scores, meaning less vulnerable neighborhoods (Norman et al. 2012a). We speculate that a possible reason for low responses from more vulnerable neighborhoods would be lack of access to Internet. This sample may under-represent some vulnerable populations in Ambos Nogales that lack access to the required technology to participate.

We compared the sample demographics to data from the US Census Bureau and the Consejo Estatal de Población (COESPO). Compared with census data, our sample population included higher percentages of women in Nogales, Arizona, and lower percentages of women respondents in Nogales, Sonora. The census population of Nogales, Arizona, is 94.6% Latino/Hispanic and 3.2% white, and the sample population is 84% Latino/Hispanic and 13% white. COESPO does not provide categorization by ethnicity (Consejo Estatal De Población, 2015; U.S. Census Bureau 2019). In both countries, our sample population included a higher proportion of people with post-graduate education (bachelor’s degrees or higher); more than 70% of survey respondents from Nogales, Sonora, attained post-graduate degrees (Consejo Estatal De Población 2015; U.S. Census Bureau 2019). Elderly (and thus potentially more vulnerable) populations are also underrepresented compared to census statistics. We hypothesize that revisions to the sampling methodology, by implementing a post-COVID electronic version of the survey, may account for these unintended biases.

To address bias, we used multiple methods, including input and validation from follow-up interviews with experts, and corroboration of our results through literature review. The combination of validation interviews and information derived from our non-random sample survey provides an assessment of flood risk perception within the Nogales watershed.

### Final interviews

We invited the same group of 36 subject matter experts from the initial interview phase to participate in follow-up interviews. We conducted 11 interviews: eight from Arizona and three from Sonora. In Arizona, federal, state, county, and municipal levels of government were represented, as well as representatives from an environmental non-governmental organization and an environmental sustainability and borderland researcher. In Sonora, both city- and state-level governments were represented. This second round of interviews was designed to verify and build off the responses to the SolVES survey. We disseminated a standardized PowerPoint presentation of the findings, including mention of potential sample biases, and then asked for their responses (Freimund 2020). The second round of interviews was conducted remotely, due to the COVID-19 pandemic. Responses for each question were compiled and analyzed for repetition and variance.

### Institutional review board (IRB)

The University of Arizona’s Institutional Review Board (IRB), an independent review committee charged with the protection of human research subjects, reviewed all research and related activities involving human subjects. The IRB approved the semi-structured interviews on September 18, 2019. The IRB approved an amendment to include the binational survey methodology on December 3, 2019.

## Results

### Question 1: what are the perceptions of flood risk throughout the binational communities of Ambos Nogales?

#### Expert perspectives of flood risk over time

All interviewees stated that they believed flood risk in Ambos Nogales has increased over time. They suggested that this increased risk stems from the aforementioned causes: population growth, lack of planning, urbanization, climate change, topography, and infrastructure (Table 3).

**Table 3:** Interviewee quotes regarding flood risk in recent decades. Adapted from Freimund (2020)

Interviewee	Quote
City actor	Definitely yes. Now there is way more pavement and, with more pavement, the water comes quicker and the risk to people increases
State actor	Yes, 100%, this is a function of population growth

#### City preparedness

Most interviewees believed that the cities of Ambos Nogales are not prepared for future floods and stated that community members may doubt city preparedness due to frequent flooding events and lack of protection from stormwater (*n* = 9). The interviewees that believed that the cities of Ambos Nogales are prepared mentioned that this may not be an issue of preparedness but one of communication. They mentioned that preparedness actions are being invested in but are not adequately communicated to the public (*n* = 2) (Tables 4 and 5).

**Table 4:** Interviewee quotes regarding city preparedness to flooding. (Adapted from Freimund (2020)

Interviewee	Quote
State actor	No [the cities] are not. So many participants believe this, because they live day to day with the failing infrastructure of Nogales. There’s sewage in the wash, failing roads. If they can’t get basic services, why would they think the city would deliver on flood control?
Federal actor	Yes, I believe they are [prepared]. They are putting in new dam areas. They are monitoring and measuring rainfall, and they’re implementing a camera system. They have data and sirens, but they need to inform people what to do when that situation happens. You can’t just scream at people and assume they’ll do the right action. We need public education about how to be prepared

**Table 5:** Summary of community flood risk perceptions from binational electronic SolVES Survey

Main theme	Key findings
Flood concern	Generally, flood concern was low throughout the watershed
	Statistically dependent relationship with previous experience of flooding (*P*-value = 0.001). Participants who experienced previous flooding in their neighborhood, generally responded with higher levels of concern
	Not significantly related to country or vulnerability level as hypothesized
Perceptions of future flood impacts	106 thought they would be impacted by floods in the future
	The largest concerns were inability to travel across town, damage to vehicles, and damage to homes
Perception of personal preparedness	Feeling personally prepared had a statistically significant relationship with gender (*P*-value = 0.017) In this sample, women generally felt less prepared than men
Perception of city preparedness	Eighty-four percent of survey respondents living in the Nogales watershed stated that they did not believe the city was prepared for future floods

*N* = 120 (AZ = 56, SON = 48, Unspecified = 16)

#### Survey results

#### Perceptions of future flood impacts

In this sample, 106 respondents believed that they may be impacted by floods in the future (mean likelihood = 3.18/5), but only 35 had previously been affected by floods in their neighborhood.

**Fig. 3:**
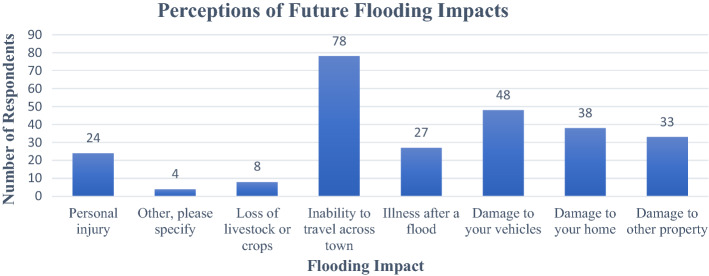
Survey responses to the question “Do you think any of the following are likely to happen to you in future floods? (choose all that apply)” (*n* = 106) We were unable to track response rate due to the electronic networking methodology used Adapted from Freimund (2020)

When asked about future flooding impacts, survey participants expressed that they feel most likely to experience an inability to travel across town, damage to their vehicles, and damage to their homes (Fig. 3).

### Question 2: how do these perceptions vary throughout the binational watershed?

We found that neither vulnerability level, as defined by Norman et al. (2012a), nor country was significantly related to level of flood concern, perceptions of future flood impacts, perceptions of personal preparedness, nor perceptions of city preparedness. In contrast, having been previously impacted by flooding showed a strong association to level of concern. Although we had hypothesized that vulnerability level would have a statistically dependent relationship with risk perceptions, this was not demonstrated in our sample. Every interviewee agreed that they would expect a lack of connection between vulnerability level and flood concern for various reasons including the recognition that people moving into highly vulnerable areas generally are not from Ambos Nogales and do not know about the flood risk (*n* = 3). They also stated that people that live in high vulnerability areas may have more immediate concerns such as access to water and proper sewage treatment (*n* = 3). Some interviewees commented that people may be more concerned about floods affecting their commute than impacting their homes (*n* = 3). Others mentioned that community members may believe that flooding is the government’s responsibility rather than the individual’s (*n* = 2).

We did find that perception of personal preparedness was significantly associated with gender throughout the binational watershed, with women feeling less prepared than men in this sample (*P*-value = 0.017). Some interviewees suggested that this difference may be due to traditional gender roles, i.e., women are more likely to be at home, taking care of children or elderly family members, which may lead to feeling less prepared. This may also signify that women would be more likely to witness the damage during a flood from their home or neighborhood (*n* = 4). Interviewees also proposed that men may be overly self-confident and believe they are more prepared than they are, which some interviewees stated may stem from *machismo* culture (*n* = 3) (Table 6).

**Table 6:** Interviewee quote regarding the relationship between personal preparedness and gender. Adapted from Freimund (2020)

Interviewee	Quote
NGO representative	We are still a very gender-based traditional culture. On both sides of the border, males are generally viewed as providers and protectors. We would see the same with other hazards or concerns.

### Question 3: what are the preferences for flood management in the binational Nogales watershed and what strategies or actions are being developed to increase resilience to flooding or to adapt to future flood risks at the municipal and binational watershed levels?

#### Community preferences for city preparedness strategies and improved flood management

Survey respondents indicated that all four of the solutions presented in the survey are important and should be implemented, with the highest percentage being flood control through new infrastructure (Table 7). The interviewees agreed with the survey respondents, stating that all the strategies suggested in Table 7 should be implemented, and that many of the strategies are being implemented by at least one stakeholder within the watershed.

**Table 7:** Summary of Survey Respondents Preferences for Future Flood Resilience Strategies and Follow-up Interview Comments. Adapted from Freimund (2020), which shows the proportion of respondents that selected each strategy as their preferred resilience option

Suggested resilience strategy	Percentage of survey responses (%)	Interviewee comments
Neighborhood awareness plans	21.37	Although most interviewees believed that neighborhood awareness plans and educational campaigns are needed, very few thought they were taking place
Flood warning systems	25.40	In Arizona, a flood warning system exists, run by Santa Cruz County. In Sonora, one is being created by the USGS, with US government funds, in collaboration with Mexican agencies
Flood control through the use of green infrastructure such as using trees and basins to slow down and reduce the water	25.00	Since the City of Green Creeks' Plan, green infrastructure projects have increased; various entities have implemented these projects including IMIP, Watershed Management Group, and university groups
Flood control through new infrastructure such as new drainage systems	28.23	Retention basins being planned in Arizona and improvements to the International Outfall Interceptor (IOI)
Total	100.00	

#### Previously implemented flood resilience strategies

When asked about previously implemented flood resilience strategies, interviewees most frequently mentioned strategic and emergency plans (*n* = 11). The interviewees discussed plans at the municipal, county, and binational level, including the US EPA Border 2020 Plan (*n* = 4) and the Arizona-Sonora Strategic Plan (*n* = 3). Frequently mentioned resilience actions included: vulnerability studies and models (*n* = 4), flood retention infrastructure, such as the gabions built in Nogales, Sonora (*n* = 4), and new systems and cameras to monitor precipitation and water levels in the wash (*n* = 3).

#### Flood resilience strategies recommended by local experts

Interviewees suggested many soft-path options to increase flood resilience in Ambos Nogales, focusing on technology sharing and local capacity building (Gleick 2003; Sovacool 2011; Wolff and Gleick 2002). They also mentioned several hard-path strategies, focusing on infrastructural solutions to flooding (Table 8; Gleick 2003; Sovacool 2011; Wolff and Gleick 2002).

**Table 8:** Summary of flood planning practitioner interview responses to the question “What is the most important thing that can be done for Nogales to become more resilient to future floods?” Adapted from Freimund (2020)

	(*n*)
*Soft path strategies*	
Raise awareness and increase educational campaigns	7
Increase utilization of green infrastructure	5
Increase regulation of building codes, trash management, and development	5
Establish community flood plans and increase community participation in flood preparedness/management	4
Share data, models, and weather information across the border	3
Build a better alarm/response system	1
Pay people a higher living wage to increase individual adaptive capacity	1
*Hard path strategies*	
Revamp existing infrastructure, such as the wash and drainage systems	5
Dig out the previously built detention basins and maintain them	4
Move the railroad	2

#### Potential for a community fund or volunteer effort

The largest group of survey respondents were willing to volunteer between 1 and 10 h/year and donate between $1–10/year for improved flood management. Most interviewees stated that educational campaigns and community green infrastructure demonstration projects would be the best ways to utilize community funds and/or to mobilize volunteers in the Nogales watershed (*n* = 9). They stated that flood education campaigns could take place in various forms, including virtual forums with communities, or with local school groups. Two interviewees mentioned that the funding could also be used to buy land for conservation, absorption of stormwater, or to build neighborhoods for relocation purposes. Interviewees also stated that should a fund exist, it would need a transparent and shared governance structure by local community members, to increase trust in the management of the fund (*n* = 3).

#### Barriers

Although the interviewees agreed that all these strategies should be implemented, including the preferred resilience strategies from the community survey, they stated that there is no centralized movement and that there are various barriers to implementing these strategies. These include: frequent change of governments, and the loss of institutional memory and momentum that accompany the change (*n* = 6), funding (*n* = 6), and temporal or procedural asymmetries in this binational setting, including overlapping jurisdictions, which complicates governance of flood management solutions (*n* = 6). Interviewees also mentioned lack of personnel (*n* = 4), lack of capacity to regulate (*n* = 3), and lack of public awareness (*n* = 3), as barriers to implementing flood resilience strategies (Table 9).

**Table 9:** Interviewee quotes regarding the barriers to implementing flood resilience strategies. Adapted from Freimund (2020)

Interviewee	Quote
NGO representative	This is a political issue at every level, city to federal to binational; it is jurisdictional and bureaucratic
State actor	[s]hifting priorities within municipal government of Nogales, Sonora. One administration might be very interested and invested in mitigating flood impacts. Then the next government might completely shift priorities. This causes loss of institutional knowledge, leadership, and progress. It feels like we’re going three steps forward, two steps back
City actor	The government is always changing; priorities change, and we lose capacity or momentum
City actor	The only way the city governments would be able to implement more is through help from the federal government. There’s just not the capacity locally

#### Collaboration

Many interviewees mentioned a history of collaboration in Ambos Nogales, particularly related to binational wastewater management, as much of the wastewater from Nogales, Sonora, is treated in Arizona. Interviewees commented that the venue for collaborating and coming to shared agreements is through the IBWC/CILA and their technical meetings (*n* = 6). Interviewees also mentioned the Arizona-Sonora Commission’s binational plans (*n* = 2) and the Border 2012 and Border 2020 groups (*n* = 3). Experts noted that collaboration has taken place on various green infrastructure projects, binational workshops and trainings, and information sharing (*n* = 3). Despite the acknowledgment that there has been a long history of binational collaboration, many interviewees commented that the binational collaborative system is unproductive or unsuccessful (Table 10).

**Table 10:** Interviewee quotes regarding the collaboration in in the Nogales watershed Adapted from Freimund (2020)

Interviewee	Quote
Federal actor	Flooding in Nogales is probably the number one hazard in all of Arizona; it would really require international cooperation and acts of Congress to fully mitigate it. Yet, there has not been much significant action to repair the situation
NGO representative	There’s not a lot of coming together, but rather blaming; lots of egos, acting like kindergarteners; this can be highlighted by the recent lawsuit between ADEQ and the IBWC

## Discussion

### Risk communication and education

Flood risk management includes communication from government entities to floodplain inhabitants to prepare for flooding and encourage adaptation to climate change (Haer et al. 2016). The results from Ambos Nogales demonstrate a call for improved flood risk communication binationally. Local experts proposed increasing educational efforts to improve resilience throughout the watershed. They also stated that if a community flood fund or other volunteer effort existed, the investment in education and communication would be most appropriate. This is particularly important in a border context, where there is a growing population in informal *colonias* (Scott et al. 2012; Norman et al. 2006, 2012a; Wilder et al. 2012); interviewees mentioned these new arrivals may be unaware of the potential risk. Attems et al. (2020) proposed that risk communication is most successful when presented by a local and trusted entity. Rollason et al. (2018) suggested that flood risk communication should include improved flood forecasting and empowering local understanding of flood impacts.

Results from our survey and interviews suggest an improving risk communication and public education about flood risk, based on this multi-pronged approach (modified from Freimund 2020):

1. To improve perceptions of personal preparedness, women should have a more active role in the design and implementation of risk communication and education initiatives (Lau et al. 2021).
2. In our study, experience with flooding events in the past greatly affected level of concern for the future. This observation is corroborated by Bradford et al. (2012), Lawrence et al. (2014), Brilly and Polic (2005), Franklin et al. (2014), and Lechowska (2018), who stated that previous experience with flooding increases risk perception and concern about future floods. Thus, it would be prudent to enlist experienced residents to relate their flooding history through a structured storytelling and risk education campaign. This idea is supported by Bradford et al. (2012), whose research recommends tailoring communication to the local context by including personal stories.
3. Public flood risk education campaigns should explain flood risk strategies that have been implemented by the city and individual actions that can take place at the household level. These education campaigns should not only focus on hard infrastructure projects completed by the city, but also raise awareness of the potential benefits from soft-path adaptation strategies that local community members and organizations could implement.
4. Develop demonstration projects with non-governmental organizations (NGOs), churches, and community groups and integrate storytelling to convey key messages. Involving non-governmental actors in these projects addresses the loss of institutional memory that results from frequent government staff turnover, which interviewees identified as a barrier to implementing resilience strategies.
5. Use the aforementioned alliances with non-governmental actors to provide a mechanism for informal cooperation and action and help create the sense of place. This strategy was highlighted by interviewees.
6. Develop improved flood warning systems as a necessary flood resilience strategy. Currently, the flood warning system consists of precipitation and stage gauges in the twin city (funded by NORCOMM), but one model could be to develop emergency warning for citizens living in the floodplain (CLIMAS 2010).

### Local flood management tools and policy

The implications for flood management are that local strategies and awareness campaigns can work equally effectively on either side of the border, regardless of different national priorities and resources. Both interview and survey responses emphasized the need for more stormwater and green infrastructure flood management tools. Infrastructure improvement, such as a new drainage system, was the survey respondents’ highest priority among the resilience strategies mentioned in the survey. Hard-path adaptations such as large-scale infrastructural improvements may increase flood resilience but are often more costly and less flexible than soft-path adaptation measures (Gleick 2003; Sovacool 2011; Wolff and Gleick 2002). These flood management strategies include the use of green infrastructure as either a demonstration and educational tool to increase awareness, or as a strategy to reduce peak flow and increase flood water absorption throughout the watershed. Most of the interviewees highlighted the need for various soft-path adaptations, which may be more affordable and include local community members and stakeholders (Gleick 2003; Sovacool 2011; Wolff and Gleick 2002). Increased regulation and enforcement were also key needs expressed by interviewees, to complement green infrastructure and reduce impervious surfaces throughout the watershed. Green infrastructure has been proposed, and plans have been developed, for the twin city area over the past 10–20 years (Norman 2005; Norman et al 2008a, b, 2010a; Viani 2019). The use of green infrastructure as a stormwater mitigation tool has been demonstrated to have significant hydrological impacts in other watersheds, and modeling in this region indicates that it could have potential to reduce runoff from flooding events (Norman et al. 2008a, b, 2010a, b; Korgaonkar et al. 2018; Pennino et al. 2016). In 2020, a proposal to develop "Sustainable Strategies for Stormwater and Combined Sewer Overflows Control in Ambos Nogales” was selected for funding by the North American Development Bank (NADBANK), a binational financial institution run by the Federal Governments of the USA and Mexico. While this study does not elaborate on specific details of water management strategies in the two cities, regarding the deployment of green infrastructure, etc., further analyses would benefit from thorough evaluation of management approaches, especially pertaining to how access to resources may affect viable strategies.

### A call for improved transboundary management and collaborative efforts

The majority of the participants in this study, both community members and local experts, did not believe that the cities of Ambos Nogales are prepared for future floods. Interviewees also mentioned that local governments do not have the capacity nor the resources to realize the suggested resilience strategies. This perception of the preparedness of local governments is similar to other areas in Mexico, as Eakin et al. (2010) suggested that flood resilience strategies may not be implemented since municipal governments are often underfunded. Lara-Valencia and Giner (2013) also stated that many border communities, such as Nogales, lag in adapting to flood risk and climate change. Temporary actions are more likely to be implemented, rather than strategies that address root causes (Eakin et al. 2010). Many local experts called for aid from state and federal governments, particularly in this binational setting and also criticized the current framework for binational collaboration, commenting that it may not be effective. Although our sample size is small, our findings portray that Federal actors were universally more optimistic about preparedness, while State actors were not (Table 4); this disconnect between the perceptions of local and Federal experts may be hindering progress in solving some of the flooding problems in Ambos Nogales.

Thus, data from this study found community support for the IBWC intention to develop a Minute for the Nogales watershed (IBWC 2017), like Minute 320 created for the Tijuana Basin (IBWC 2015). The Binational Core Group and smaller Working Groups of stakeholders created in Minute 320 aimed to collaboratively set priorities, execute projects, and conduct community outreach relating to transboundary water projects including flooding, wastewater, erosion, and climate change (IBWC 2015). Focusing a proposed Nogales watershed Minute on a flood-related binational working group would improve the effectiveness of binational efforts—an issue raised by several stakeholders—and it would provide a foundation for improved trust and shared responsibility for developing and implementing green infrastructure and other stormwater runoff resilience solutions. As Wilder et al. (2020) claimed, to address issues relating to water and climate in the US–Mexico border, the IBWC Minute process allows for flexibility while still maintaining the formal binational cooperative framework. A new Minute could help strengthen binational collaborative networks, draw Federal attention to the problem, and create the infrastructure for those networks to distribute resources to address flood related issues, which would contribute to the adaptive capacity of Ambos Nogales (Wilder et al. 2012, 2013).

## Conclusions

A survey was used to identify key aspects of perceptions of flood risk and potential actions to bolster resilience in Ambos Nogales, twin cities straddling the US–Mexico border, that offer insights for policy and practice. Our results showed few differences between nations in terms of flood risk perception and preparedness. Regarding perceptions of flood risk, people in the cities of Ambos Nogales were generally more concerned about the risks of traveling across town during floods, and damage to their vehicles, than about impacts to their homes or neighborhoods. Globally, it is being acknowledged that gender plays a key role in how people experience and perceive climate change (Enarson 2012, 2014; Enarson et al. 2018; Lau et al, 2021). This was true in our study, where gender was strongly associated with feelings of personal preparedness. Other studies demonstrate that women are more at risk during and post-natural disasters, and thus, the heightened risk perception of women in Ambos Nogales may be due to increased barriers to overcome during flooding events (Enarson 2012, 2014; Enarson et al. 2018; Lau et al, 2021). Hence, binational administrators could utilize our results to make changes to improve gender equity in decision making about flood risk management. We also recommend additional research to assess the relationship between gender and flood preparedness and potential strategies to better incorporate gender equity into policies and projects that address flooding in Ambos Nogales in a way that reduces barriers for women to participate. In response to variance across the watershed, geographical indicators didn’t have a statistically significant relationship with flood risk perception but instead was associated with previous flood experience, and to a lesser extent, gender. Flood concern and perception of future flood risk were not significantly related to either country or vulnerability level. This was a surprising finding and an important contribution, especially because the impacts of flooding are more physically apparent in the more vulnerable neighborhoods vs more affluent and in Mexico vs. the USA (Norman et al. 2010a, 2012b, c). Interestingly, the majority of local survey and interview respondents did not believe that either of the cities are prepared, but the Federal actors did. This may be part of the problem of getting Federal dollars toward addressing flooding in Ambos Nogales. Finally, in response to preferences or strategies to increase resilience, an overwhelming response supported green infrastructure and flood risk awareness campaigns.

Participants in this study also proposed including green infrastructure (such as basins or artificial wetlands), new stormwater infrastructure (such as drainage systems), improved emergency warning systems, and regulations and enforcement of codes to reduce the expansion of hard surfaces in the watershed. Barriers cited were funding, asymmetrical governance structures, rapid government change, lack of personnel and local capacity, as well as difficulties collaborating between various jurisdictions and priorities. Most of the interviewees encouraged efforts for increasing flood risk awareness through education and green infrastructure demonstration projects; future studies could examine the efficacy of these actions and whether they influence residents’ concern and preparedness for future floods and expressed willingness to volunteer or donate money to help. Data and studies, maps, and video monitoring were suggested to increase preparedness and flood early warning efforts and inform flood resilience planning and practice.

The iterative engagement approach, garnering perceptions of both local experts and public participants, is a valuable methodology that allowed the facilitator to both convey survey results to decision makers and also give them multiple chances to think through barriers and opportunities for binational flood management. Our findings suggest clear directions for outreach and public education, to inform awareness of flood risk and preparedness for floods. Coordination of demonstration projects, flood preparedness and risk awareness campaigns with NGOs, church groups and other community groups would foster informal flood governance and help maintain momentum, even when policy priorities change as result of the frequent turnover in city and state governments. Specifically, based on the binational San Diego–Tijuana watershed and the political movement to include Minute 320, the Ambos Nogales watershed would likely benefit from a similar effort to generate trust in government measures, to reduce risk, and to inform the risk perceptions of residents. Many participants in this study noted that floods are a binational issue and stated that the cities lack the capacity and funding to implement needed resilience strategies (Mumme 2020; Pineda Pablos et al. 2020).
